# Supplementary material for: Are carbon emissions trading and green financial instruments synergistic? -Comprehensive quantitative research based on content analysis
Source: PLoS One. 2024 Mar 7;19(3):e0298601. doi: 10.1371/journal.pone.0298601 (PMC10919601; doi:10.1371/journal.pone.0298601)
Supplement: S1 Data — (ZIP) [file pone.0298601.s001.zip › Data/地级市Z_F工作报告.zip!/║■▒▒/╢⌡╓▌╩╨/└┤╫╘╣1⁄2╓┌║┼í╛┬φ┐╦╩2╛▌═°í┐_2006.docx]

各位代表：

　　现在，我代表市人民政府向大会作《政府工作报告》，请连同《鄂州市国民经济和社会发展第十一个五年规划纲要（草案）》一并审议，并请市政协各位委员和列席人员提出意见。

　　一、“十五”时期经济社会发展及2005年工作的回顾

　　“十五”时期，在省委、省政府和市委的正确领导下，全市人民以邓小平理论和“三个代表”重要思想为指导，牢固树立和落实科学发展观，始终坚持以经济建设为中心，抢抓机遇，求是创新，扎实工作，克难奋进，成功应对非典疫情和禽流感等自然灾害挑战，有效克服了经济运行和社会发展中出现的各种困难，全面或超额完成了“十五”计划确定的各项目标任务。

　　综合实力迈上新台阶。过去的五年，是我市经济规模不断扩大、发展速度不断加快、经济质量不断提高的五年。经济总量大幅增长。2005年全市完成地区生产总值146.97亿元，比2000年增长63.9%，年均增长10.4%；人均生产总值13908元，比2000年增长43.9%，年均增长9.5%。工业经济快速增长。2005年，全市规模以上工业企业完成增加值60.89亿元，比2000年增长107.0%，年均增长15.7%；实现产品销售收入161.5亿元，比2000年增长60.5%，年均增长9.9%。高新技术产业发展迅速，2005年完成增加值12.2亿元，比2000年增长95.7%，年均增长14.4%。农村经济稳步发展。2005年完成农业增加值23.7亿元，比2000年增长44.9%，年均增长7.7%，水产、蔬菜、畜牧、林果已成为我市农业的重要支柱产业。五年来，农业综合开发累计投入4099.4万元，改造中低产田10.57万亩。水利基础设施建设共完成投资7.66亿元，加固长江干堤66.7公里、内湖堤防210公里、35座水库整险达标。打工经济发展迅速，已成为农民增收致富的重要途径。第三产业发展势头良好。通过引进国内知名商贸企业，盘活了一批国合商业资产存量，提升了鄂州消费档次。房地产业迅猛发展，五年累计完成投资16.02亿元，比2000年增长3.9倍，年均增长34.0%。住房公积金管理走在全省前列。成功举办了三届武昌鱼国际文化旅游节和五届梁子湖捕鱼旅游节。我市荣获“中国优秀旅游城市”称号，对外形象和知名度得到提升。金融保险业健康发展，信贷质量不断提高。财政收入连创新高。继2004年突破10亿元大关后，2005年全市财政收入达到13.3亿元，比2000年翻一番，年均增长16.8%，其中：地方一般预算收入达到4.93亿元，比2000年增长89.1%，年均增长13.6%。国地两税收入占财政收入的比重达到91.3%，比2000年提高了17.2个百分点。经济外向度不断增加。“十五”期间全市完成招商引资总额10.07亿美元，年均增长12.2%；2005年全市外贸出口总值完成2.95亿美元，比2000年增长74.3%，年均增长11.8%，葛店高新技术开发区、花湖民营开发区、梁子湖生态旅游区、红莲湖旅游度假区等园区建设进展顺利，区域合作不断加强，融入武汉城市圈步伐加快。重点项目建设明显加强。“323”工程建设顺利。“十五”期间累计完成固定资产投资198.3亿元，比“九五”期内增加70.4亿元，年均增长14.3%。总投资均为12亿元的鄂钢冷轧板工程和武钢鄂州球团厂项目成为我市经济增长的新亮点。

经济体制改革实现新突破。“十五”期间的改革所涉及的范围、推进的力度、取得的成果引人瞩目。国企“两改”稳步推进。经过五年的努力，列入市级改制计划的131家企业，已批复改制方案130家，安置职工40556人。以吴城钢铁公司为代表的一批改制企业的快速发展，充分证明了我市国企“两改”是成功的。国企“两改”的成功，使我市民营经济的发展跃上了一个新的台阶。2005年全市拥有民营企业2263家，比2000年增加1318家，五年来平均每年增加264家。2005年全市民营经济完成的生产总值、上交的税收在全市总量中分别占58.1%和55.6%。农村税费改革取得重大突破。2005年全面取消了农业税，在我市结束了几千年农民种田交税的历史。五年来，全市农民负担总额减少了9552.9万元。财政体制改革不断深化。全面推行部门预算、国库集中收付、政府采购、收支两条线四项制度改革，在全省财政改革与管理综合考评中，连续2年名列全省第一。行政事业单位改革收效明显。“十五”期间，市、区、乡（镇、街办）三级行政机构和事业单位得到精简，富余人员得到分流，工作效率得到提高。二医院民营化改革取得成功。粮食、商贸、供销、教育、科技、文化、卫生等各行各业的改革进展顺利。

　　城市品味得到新提升。“十五”期间，城市建设力度不断加大，制订了《鄂州市城镇化发展战略》，修编了《鄂州市城市总体规划》，明确了“一主三辅”的城市发展总思路，确立了建设山水园林城的总目标。五年来，城市基础设施累计投资9.66亿元，是“九五”期间的2.98倍。中心城区建成区面积达31平方公里，比“九五”期末净增6平方公里；鄂黄大桥、凤凰广场、南浦虹桥、鄂州高中、广电大楼、迎宾大道、沿江大道等标志性工程为城市增添了新的亮点。洋澜湖综合治理初见成效，水体恶化得到有效遏制，破堤还湖效果明显，湖面面积由整治前的2300亩恢复到6009亩。小城镇建设步伐加快，城镇化率达53.6%，比“九五”末增加7.4个百分点。城区新增绿地面积6000亩。城市管理力度加大，市容市貌得到较大改观。环境保护工作进一步加强，五年共审批建设项目546个，大中型建设项目“环评”、“三同时”执行率达100%，我市被列为全国生态示范试点城市。国有土地资源管理得到加强。交通建设累计投入8.2亿元，比“九五”时期增长121.6%，鄂黄大桥一级公路连接线、316国道改造、梁子湖码头及航道疏浚等重大工程相继竣工。公路密度每百平方公里达124.3公里，被列为全省公路建设示范市。村村通公路达1100公里，农村公路建设走在全省前列。城乡电网改造累计投入5.93亿元，新建变电站34座，实现了城乡同网同价。

　　城乡居民生活达到新水平。“十五”时期，我市经济持续发展，群众消费结构发生新的变化，人民生活水平得到较大提高。2005年城镇居民人均可支配收入达到8187元，年均增长8.12%；农民人平纯收入3495元，年均增长7.02%。城乡居民住房条件明显改善，2005年全市城镇人均居住面积28.7平方米，农村人均住宅面积43平方米，分别比2000年增长55.3%和29.9%；天然气工程顺利实施，13000户居民用上了清洁、高效能源。2005年全社会商品购买力达到62.1亿元，比2000年增长66.9%，年均增长10.8%。人均食品消费支出占人均消费总支出41.1%，处于国际公认的小康生活水平区间。物价水平总体稳定，居民消费价格指数年年控制在国家规定范围以内，圆满完成了省政府下达的价格调控目标任务。

民主法制建设有了新进展。“十五”期间，政府依法行政意识进一步增强。五年来，共办理人大代表、政协委员的建议和提案1443件，满意率达到98.76%。全面推行政务公开，政府工作透明度和市民对社会公共事务的参与度不断提高。认真贯彻执行《行政许可法》，行政许可项目由申报的1063项审减到310项，确认许可收费项目37项，审减了362项。源头治腐力度不断加大，成立了行政服务中心、综合招投标中心、经济发展投诉中心、国库集中收付中心和政府采购中心，从制度和程序上加强了源头治腐工作，审计、监察工作得到加强。社会治安综合治理形势进一步好转，全民法制宣传教育深入进行，“四五”普法成效良好。

　　社会事业呈现新局面。“十五”以来，在促进经济快速增长的同时，十分注重社会事业的协调发展。全市共实施国家、省科技计划项目228项，申报专利211件，获得省科技进步和科技创新奖18项。“普九”成果进一步巩固，教育质量全面提高。基本消除农村中小学D级危房。邮政、通讯、广播事业发展迅猛。群众文化体育活动蓬勃开展，“周周乐”广场文化形成品牌。进一步加强了公共卫生体系建设，成功应对“非典”、禽流感等重大疫情，城乡医疗条件逐步改善，艾滋病、血吸虫病、地方病防治工作得到加强。低生育水平持续稳定，“十五”期间人口出生率为9.09‰，低于省定责任目标。社会保障工作进一步加强，离退休人员养老金、下岗职工基本生活费按时足额发放，符合条件的城镇贫困居民，全部纳入了最低生活保障范围。就业再就业工作稳步发展，2005年城镇登记失业率为4.12%，低于全省4.3%的目标控制数。2005年筹资670.3万元，帮助30个重点贫困村整村推进扶贫工作初见成效。认真实施了农村五保户供养的“福星工程”。安全生产、质量技术监督和食品、药品、烟草市场整顿等逐步加强，杜绝了重特大安全事故的发生。国防教育、征兵和民兵预备役工作不断加强，军政、军民关系进一步密切。我市先后被评为省级文明城市、卫生城市、园林城市、双拥模范城。统计、信访、对台、外事侨务、人防、民族宗教等各项工作取得新成绩，档案、方志、气象、残联、老龄等事业有了新发展。

　　2005年我们坚持以科学发展观统揽经济社会发展全局 ，保持了经济社会协调发展的良好态势，圆满完成了五届人大二次会议上提出的各项目标任务。10大经济指标均完成或超额完成，项目建设的“323”工程、城市建设“6436”工程、交通建设10大工程顺利完工或开工。获得了湖北省城镇规划建设管理“楚天杯”。在此，特别需要向各位代表报告的是，在五届人大二次会议上市政府向全市人民承诺的8件实事已全部兑现，8件实事的总受益人口在10万人以上。与此同时，还需要向各位代表报告的是在2005年全市经济工作中作出重大贡献的10大纳税企业是鄂钢、鄂州电厂、鄂州程潮铁矿、市供电公司、市吴城钢铁公司、市烟草公司、市波尔亚太公司、市科氏沥青公司、市华明实业公司、市龟山铁矿，其中：当年增幅超过翻番目标的有市龟山铁矿、市科氏沥青公司、市烟草公司、鄂州程潮铁矿。

　　各位代表，过去的五年，是我市经济持续快速发展、综合实力显著增强的五年，是城乡面貌发生巨大变化、人民生活水平进一步提高的五年，是改革开放取得重大突破、经济发展活力不断增强的五年，是三个文明协调发展、各项社会事业全面进步的五年。总结过去五年的政府工作，有六条基本经验值得在今后的工作中借鉴。一是必须千方百计促进经济又快又好地发展。发展是解决鄂州一切问题的关键，只有始终高举发展的大旗，保持较快的发展速度，才能在工作中掌握主动；只有正确处理速度与效益、规模与质量、当前与长远的关系，才能实现经济持续协调健康地向前发展。二是必须把改革作为推动经济社会发展的强大动力。只有站在时代的前列，准确地把握时代的脉搏，不失时机地革除影响发展的各种弊端，不断地创新体制机制，才能确保经济社会发展的活力不减、动力不竭；只有把改革的力度和人民群众可以承受的程度有机地统一起来，营造稳定的社会环境，确保各项改革顺利达到预期目标，才能促进经济社会的和谐发展。三是必须坚定不移地走新型工业化之路。鄂州之所以“立市”，是因为工业基础较好，鄂州要“强市”，还得不遗余力地发展工业。实践告诉我们：没有工业化，就没有鄂州的现代化。只有坚持走新型工业化道路，才能通过工业的超常发展带动经济的整体跨越。四是必须将改善发展环境作为政府领导经济工作的着力点。只有牢固树立抓城市建设就是抓经济建设、抓城市发展就是抓经济发展的理念，高水平经营城市，高质量完善基础设施，不断提升城市品位，才能凝集人气，激发投资热情；只有下大力气改善服务环境、治安环境，为市场主体创造优质舒适的营商条件，才能最大限度地吸纳生产要素，让创造财富的源泉充分涌流。五是必须认真解决事关群众切身利益的突出问题。只有牢固树立执政为民的思想，坚持发展为了人民，发展依靠人民，发展成果由人民共享，把解决事关群众切身利益的突出问题作为政府工作的重要内容，尽力做好城乡弱势群体救助、就业再就业、农业农村基础设施建设、扶贫开发、生态环境保护等工作，才能进一步密切党和政府与人民群众的联系。六是必须不断提高政府驾驭经济社会发展的能力和水平。政府的能力和水平影响着一个地方经济社会发展的速度和效益，经济社会越是发展，对政府的能力要求越高。不断提高政府在复杂环境中驾驭全局的能力、在经济发展中把握机遇的能力、在社会进步中统筹各方的能力，是过去五年的一条重要经验。实践证明：只有不断地加强政府的能力建设，才能在新的历史条件下实现经济社会又快又好的发展。

　　各位代表，“十五”的成绩来之不易，凝聚着全市人民的智慧和心血。借此机会，我代表市人民政府向全体代表、政协委员，向全市各条战线的广大干部和群众、离退休老同志，向驻市的解放军、武警和消防官兵，向各民主党派、工商联、无党派人士、人民团体，向关心支持我市建设的港澳台同胞、海外侨胞、国际友人表示衷心的感谢！

　　在充分肯定成绩的同时，我们也清醒地看到，当前经济社会生活中还存在着不少矛盾和问题：一是经济规模小，结构调整任务重。2005年我市地区生产总值、地方一般预算收入两项指标绝对数在全省市州中排名靠后，与鄂州地级市的地位仍不相称。从经济结构上看，主要表现为市属地方企业与中省企业发展不平衡、一二三产业发展不平衡、轻重工业发展不平衡，由于上述“三个不平衡”又带来了产业结构与就业结构不协调、地方一般预算收入与财政收入总量不协调、经济总量增长与居民收入增加不协调，“三个不协调”直接表现为就业不够、地方可用财力不足、城乡居民购买力有限的“三个难题”上。二是创新能力不强，发展后劲不足。创新平台搭建不够，创新要素整合不力，创新机制激励不足；高新技术产业块头不大，科技对经济增长贡献率不高；市场主体培育不够，企业活力不足；社会和企业储备项目不多，可选择余地不大；经济增长方式有待改进，发展后劲有待增强。三是农业“龙头”带动不力，产业化程度不高。这一问题集中表现为“三个缺少”，即缺少大企业带动，企业与农户难以结成真正的利益共同体；缺少大基地带动，规模和特色优势不突出；缺少大品牌带动，资源优势不能完全转化为经济优势。四是统筹发展任务重，社会保障压力大。在城市快速发展的同时，农村建设相对滞后；在内源型经济较快发展的同时，外向型经济发展缓慢；在经济较快增长的同时，社会事业发展仍待加快；在千方百计扩大就业、增强社会保障能力的同时，对弱势群体的救助任务仍然艰巨。与此同时，政府工作中还有一些不足，政府自身改革和建设滞后，社会管理和公共服务职能履行不到位，少数部门办事效率不高，还存在官僚主义、形式主义和腐败现象。这些问题已经引起了我们的高度重视，今后将采取积极的措施，努力加以解决。

　　二、“十一五”时期经济社会发展的主要目标和任务

　　实现鄂州经济社会又快又好地发展，全面建设富裕型小康社会，是“十一五”规划的立足点和出发点。根据市委《关于制定国民经济和社会发展第十一个五年规划的建议》，市政府在调查研究的基础上起草了《纲要（草案）》，已提交到这次大会审议。

　　“十一五”期间我市国民经济和社会发展的指导思想是：以邓小平理论和“三个代表”重要思想为指导，以科学发展观统领经济社会发展全局 ，全面落实党的十六届五中全会精神，紧紧抓住实施中部崛起战略和建设武汉城市圈的发展机遇，着力实施工业强市、科教兴市、开放活市和可持续发展战略，走经济发展、生态良好、社会和谐的科学发展之路，力争使鄂州成为武汉城市圈内优势明显、极具活力、快速发展的增长极，为全面建设小康社会打下坚实基础。“十一五”期间，我市国民经济和社会发展的总体目标是：在结构优化、效益增长的基础上，力争地区生产总值年均增长12%，财政收入年均增长15%，2010年比2005年翻一番；城镇居民人均可支配收入年均增长8%，农民人平纯收入年均增长6.5%；城镇登记失业率控制在4.5%以内，万元生产总值综合能耗比“十五”末降低20%以上。到“十一五”末使我市经济、政治、文化、社会全面发展，经济实力大幅增强，民主建设更加完善，社会事业全面进步，人民生活更加殷实，和谐鄂州建设取得新的更大成效。

　　根据上述指导思想和发展目标，《纲要（草案）》明确提出“十一五”期间经济和社会发展的任务及措施，主要包括以下八个方面：

　　（一）扎实推进“工业强市”战略。率先发展工业 ，是鄂州现实基础所决定的；科学发展工业，是鄂州长远需要所决定的。必须扎实推进“工业强市”战略，切实走新型工业化之路，坚持节约发展、清洁发展、安全发展，使经济步入全面协调可持续发展的轨道。

　　一是全力支持大中型企业。鄂钢、鄂州电厂、武钢鄂州球团厂等大中型企业运行正常与否，直接关系到全市经济增长的快慢。只有一如既往地对大中型企业的发展倾注心力，“工业强市”的发展战略才可能取得明显实效。发挥鄂钢与武钢重组的优势，支持鄂钢实施冷轧板、中厚板等重大项目建设，不断调整产品结构，扩大生产能力，提高经济效益。支持武钢鄂州球团厂二期建设，尽快形成年产1000万吨球团生产能力。加快发展能源产业，确保鄂州电厂二期工程并网发电。不断提高产业配套能力，有针对性地扶持一批中小企业研发生产与大中型企业配套的上下游产品，使大中型企业能够就地配套，降低生产成本，拉长产业链条，促进产业聚集。

　　二是改造提升传统产业。进一步淘汰落后生产工艺，提高产品科技含量，走经济效益好、资源消耗低、环境污染少、人力资源优势得到充分发挥的新型工业化之路。抓紧实施吴城钢铁百万吨技改工程，提高产品附加值；支持世纪新峰、华新鄂州公司采用先进的设备和技术，推广新型生产工艺；以鄂重、富升锻压等优势企业和白浒镇工业园为载体，提高环保机械、锻压机械、精密模具、精密铸件等产品的科技含量和信息化水平；支持多佳、枫树发展全光纯涤纶线、休闲服装，全面提升产品质量。

　　三是加快发展高新产业。进一步采取措施，加大扶持力度，促进高新技术产业加快发展。做足做活“依武而昌”文章，充分借助武汉的科教优势，用更有力的措施、更优惠的政策吸引高等院校、科研院所的科研项目到鄂州落户、科技人才到鄂州创业；以葛店开发区为依托，发展壮大医药产业，继续引进一批带动力强、辐射力强的生物医药项目，使“中国药谷”成为真正的园区品牌、产业品牌、招商品牌；主动接受“武汉?中国光谷”的辐射，大力发展电子信息产业，积极开发武汉80万吨乙烯项目配套产品、玻璃基微硬盘、计算机外围产品和应用软件等产品。

　　（二）加快建设社会主义新农村。建设“生产发展、生活宽裕、乡风文明、村容整洁、管理民主”的社会主义新农村是“十一五”时期的一项重要任务，也是我市实现城乡统筹发展的一次重大机遇。作为全省经济发展较快、城镇化水平较高的中等城市，在社会主义新农村建设方面理应走在全省前列。

　　一是党的政策进农村。进一步贯彻“多予、少取、放活”的方针，继续减轻农民负担，防止农民负担反弹；进一步探索工业反哺农业、城市支持农村的途径和办法，促进农业增效、农民增收和农村发展；进一步贯彻农村土地承包法，依法稳定土地承包关系，切实保护农民土地承包权益。积极开展乡村债务清理，逐步化解老债，避免新增债务；培育农村新型社会化组织，鼓励和支持农民在生产、加工、流通等环节组建多种形式的专业合作经济组织。在贯彻实施各项经济政策的同时，全面落实党和政府关于加快新农村建设的政治、文化和社会发展的各项政策。采取多种措施，把政策宣传到农村、落实到农村，建立和完善农民直接享受党和政府各项惠农政策的长效机制。

　　二是城市规划进农村。建设社会主义新农村，必须统筹城乡规划，只有将村镇建设纳入城市建设的总体框架，才能达到“乡风文明，村容整洁”的目标要求。村镇规划必须充分挖掘历史资源、文化资源、自然资源，突出乡村特色、水乡特色、地方特色。按照生产生活、工业农业、经济社会发展的功能分区要求，认真制订村庄建设和人居环境治理的指导性目录，重点解决农民在饮水、行路、用电和燃料等方面的困难，凡符合目录的项目可给予资金、实物等方面的引导和扶持。切实加强总体规划的控制和管理，重点抓好宅基地规划和管理，有计划、分步骤迁建居住分散的自然村庄，节约村庄建设用地，向农民免费提供经济安全适用、节地节能节材的住宅设计图样，防止随意搭建、杂乱无序的现象发生。引导和帮助农民切实解决住宅与畜禽圈舍混杂问题，做好农村污水、垃圾和厕所治理。

　　三是产业资本进农村。建设社会主义新农村，不能就农村论农村，要跳出“三农”抓“三农”，创造条件让产业资本进农村，在农产品转化增值、农业产业化上做大文章。通过优惠政策引导社会资金投向农产品加工等薄弱环节，提高我市农产品加工转化增值率。选择一批基础好、带动能力强的农产品加工企业，从资金、技术、人才等方面给予重点扶持，着力提高其科技创新能力、市场竞争力和辐射带动能力。调整农业产业结构，发展优质、高产、高效、生态、安全的城郊型农业。加快优质粮油基地建设，培植壮大水产、蔬菜、畜牧、林果四大支柱产业。通过龙头企业和特色基地的带动，拉长加粗农业产业链条，做优做强“武昌鱼”、“梁子湖（岛）”等品牌，使资源优势变为产业优势、产业优势变为经济优势。

　　四是公共产品进农村。按照“全面规划、统筹兼顾、标本兼治、综合治理”的要求 ，以江河（湖）无病堤、山上无险库、平原无渍涝、低凹（山冲）无冷浸为方向，加快农业基础设施建设。市政府决定用三年左右时间筹资3900万元对排涝55千瓦、抗旱30千瓦的农村泵站进行改造，同时配套进行渠道维修和建设。积极争取中央、省里对农村公路网建设的支持，在村村通公路的基础上实现自然村通公路。进一步完善电力供应体系，加快自来水管网建设和农村改厕等工作步伐。在做好规划、办好试点的基础上，全面启动新农村建设。用三年时间筹资1500万元，帮助全市30个贫困村脱贫致富。巩固提高“普九”成果，落实义务教育阶段学生免收学杂费等各项优惠政策。改善农村公共卫生条件，完善医疗服务体系，建立重大疾病医疗救助制度和新型农村合作医疗制度，切实解决农民看病难、看病贵问题。通过政府的主导作用，加大公共财政向农村转移力度，促进城市基础设施向农村延伸，城市公共服务向农村覆盖，城市现代文明向农村辐射。

　　五是科技文化进农村。健全农业科技推广体系，探索和完善基层农技推广服务体制和机制，推动农业科技进村入户。采取科技培训、示范、宣传、技术承包和办科技联系点、示范户等多种形式，大力推广新品种、新技术、新模式、新农业、新机械、新工艺。加大对农村文化事业的投入，探索建设农村先进文化的新路子。以政府为主导，以乡镇为依托，以村为重点，建设区、乡镇村文化设施和文化活动场所。加强农村广播电视建设，实现有线电视“村村通”。开展多种形式的群众文化活动，进一步丰富农村文化生活。开展创建“富在农家、学在农家、美在农家、乐在农家”活动，使农民在物质生活富裕的同时实现精神生活富裕。

　　（三）切实增强自主创新能力。提高自主创新能力是调整经济结构和转变经济增长方式的中心环节，是突破资源要素制约和缓解环境压力的根本途径，是促进产业升级和提高企业竞争力的关键举措。“十一五”期间一定要把自主创新放到更加突出位置，力争在一些重要领域取得突破。

　　一是突出创新主体。引导企业增加科技投入，加强企业技术开发中心建设，注重引进技术的二次创新。实行支持企业自主创新的财税、金融和政府奖励政策，强化科技经费对自主创新的引导和保障作用，激发中小企业的创新活力。积极培育和发展科技中介服务组织，加快科技孵化器、中试基地、技术交易中心等创业服务体系建设，推动科技中小企业的发展。

　　二是构建创新平台。高水平的工业园区，是自主创新的重要平台和载体。完善园区建设思路，引进知名的园区开发商和服务商对重点园区进行包片开发，使园区建设和管理符合国际通行的标准和规范，更好地实现承接科技资本转移的功能。加快园区水、电、路、通讯等基础设施建设步伐，完善园区服务功能，使之成为集聚创新要素的“洼地”。葛店高新区要发挥独特优势，抓住国家级生物医药产业基地和“两谷”高新技术产业带建设的机遇，用优良环境、优质服务、优厚回报吸引“光谷”科研成果进区中试、转让、应用，使葛店高新区成为武汉城市圈电子信息技术的孵化基地，成为武汉科技成果的转化基地，成为推动全市自主创新能力不断增强的研发基地。

　　三是培养创新人才。创新人才是鄂州最稀缺的资源。不断地营造鼓励创新、宽容失误的社会氛围，鼓励科技工作者放心、放手、放胆进行技术攻关。充分发挥市场在人才配置中的基础作用，建立一个培养人才、选拔人才、吸引人才的良好机制。大力发展职业教育，抓紧培养急需的技工人才和农村乡土人才。通过优惠政策引进经济和社会发展急需的高层次专业技术人才和高级管理人才，使鄂州成为群贤荟萃的“人才高地”。坚持用事业凝聚人才，用实践造就人才，用机制激励人才，用法制保障人才，努力形成创新人才辈出的局面。

　　（四）不断提高对外开放水平。坚持引进来、走出去相结合，充分利用国际国内两个市场、两种资源，努力适应对外开放的新形势，不断拓宽对外开放的新领域。

　　一是加大招商引资力度。牢固树立开放优先、企业优先的思想观念，坚持工作向招商倾斜、精力向项目倾斜、服务向基层倾斜。完善招商引资的方式方法，突出区位优势和环境优势吸引项目，突出产业优势和市场优势吸引客商，突出资源优势和技术优势吸引投资。鄂城区可进一步利用自身资源和区位优势，吸引外商参与开发；华容区可发挥紧邻武汉的优势，借梯登高，借船出海，实现招商引资的突破；梁子湖区可利用非金属矿和旅游资源，拓展招商引资领域。积极探索基础设施建设方面的招商引资，扩大商贸、金融、旅游运输、信息咨询等服务领域的利用外资规模。

　　二是优化出口商品结构。坚持以质取胜战略，增强出口产品的市场竞争力。重点抓好名优轻纺产品、具有优势的机电产品以及高新技术产品出口，提高农副产品出口比重，进一步优化出口商品结构；提高自营出口企业、外资企业的出口份额，对出口大户继续实行挂点联系责任制。巩固欧美日及东南亚出口市场，加大对拉美、非洲以及中东等新兴出口市场开拓的力度；加强口岸基础设施建设，争取开放鄂州二类水运口岸。

　　三是实施“走出去”战略。坚持立足鄂州发展鄂州和跳出鄂州发展鄂州并举，支持和引导优势企业到市外、境外投资办厂，开展加工贸易和资源开发，更多地利用国外的资源和市场。鼓励和支持企业开展对外工程承包与劳务合作，创新合作方式，扩大合作规模。多方搜集劳务供求信息，加强劳务输出，为农村剩余劳动力找出路，为下岗职工再就业服务。拓宽与国外友好城市的交流渠道，扩大交往深度和广度，实现经济、科教、文卫等全方位合作。

　　（五）继续深化各项改革。改革已进入攻坚阶段，涉及的范围更广、矛盾更多、困难更大、利益关系更复杂，必须以科学发展观为指导，以更大的决心坚定不移地推进改革。

　　一是继续深化企业改革。坚持以民营化为基本取向，全面完成国企改革任务。以完善法人治理结构为重点，加快建立规范的现代企业制度。以优势企业和名牌产品为依托，通过生产要素的优化配置，努力形成一批拥有自主知识产权、主业突出、核心竞争力强的企业集团。不断改善创业环境，培育民营经济主体，改变我市民营企业规模过小的局面。积极建设创业辅导、融资担保、协会商会等服务体系，简化民间投资准入条件和程序，降低民营企业的交易成本，促进民营经济大发展。

　　二是深化行政事业单位管理体制改革。推进政府职能转变，必须在体制机制上改进政府及其部门管理经济、社会的方式和手段，重点解决工作中越位、缺位、错位问题。进一步清理政府和部门的各种政策规定，减少审批程序，减化各种手续，减轻业主负担，提高办事效率。坚决纠正行政审批过程中的不作为、乱作为和慢作为等问题，坚决防范国家利益部门化、部门利益集团化、公共利益私有化等严重违法违纪现象。稳步推行以乡镇机构、农村义务教育、乡村卫生体制和区乡财政体制改革为主要内容的农村综合配套改革，切实转变乡镇政府职能，精简机构和人员。加快教育卫生等事业单位改革，有效整合教育卫生资源，努力解决“因学致贫、因病致贫”的问题。

　　三是深化财政金融体制改革。继续深化财政四项改革，完善财政转移支付制度，加快建立公共财政体系。切实规范政府投资行为，合理界定政府投资职能，健全政府投资决策制度。积极支持商业银行改革，引进发展多种所有制的金融企业。加快农村信用合作社改革，完善农村小额信用贷款和农户联保贷款制度，增强金融对农村经济发展的支持力度。

　　（六）着力优化发展环境。加快鄂州发展，关键是要有一个好的发展环境。“十一五”期间，政府统领经济发展的重点将放在改善发展环境上。

　　一是加强城市设施建设。围绕建成国家级园林城市的目标，坚持高起点规划、高标准建设、高水平管理城市，不断强化功能、增强实力、提升品位，提高对市民的归属感、对外商的吸引力。树立经营城市的理念，建立多元投融资机制，广泛吸纳社会资金投入城市建设。根据“一主三辅”的城市发展总 思路，主城区逐步建成为环境优美、设施完善、生态和谐、市场繁荣、特色鲜明的山水园林城；辅城区按照突出特色、统筹发展的要求，逐步成为所在区域的经济、政治、文化、社会活动中心，成为以工促农、以城带乡的“交汇点”。按照建立和完善综合交通运输体系的要求，重点兴建鄂州至武汉快速通道、东葛大道、葛湖路等重点工程，开通鄂钢、武钢鄂州球团厂至长江码头的铁路联线，建设五丈港码头二期工程，筹划建设武汉至鄂州轻轨，加快农村自然村水泥公路建设。

　　二是加快改善投资环境。坚持不懈地营造规范严明的法制环境，认真贯彻实施行政许可法，做到依法办事，公正执法，杜绝对外商、外来投资企业的歧视。加强经济环境投诉中心建设，严厉查处各种执法不公、损害经济发展环境的行为，保护投资者利益。继续实施企业“宁静日”制度，使其逐渐成为我市招商引资的一块牌子。坚持不懈地营造诚实守信的信用环境，加快建立与现代市场经济相适应的社会信用体系，进一步加大信用创建力度，打击逃废金融债务行为，增强全社会的诚信意识，使守信者致富，失信者在市场上无立足之地。坚持不懈地营造周到优良的服务环境，重点发展以旅游业、金融保险业、信息业和现代物流业为主的第三产业，不断改善我市产业服务环境。严厉打击扰乱市场秩序的各种违法犯罪行为，努力为各类市场主体提供周到、便捷、优质、高效的服务。

　　（七）建设资源节约型、环境友好型社会。建设资源节约型和环境友好型社会，是落实科学发展观的重要内容，是提高发展质量和效益的根本途径，对 产业偏重、能耗偏高的我市来讲，尤其显得重要而迫切。

　　一是推进节约资源的生产方式和消费模式。坚持资源保障与节约利用并举，把节约放在首位。根据经济社会发展的需要，搞好土地、能源、资金和水资源的统筹安排和优化配置，杜绝资源浪费，降低资源消耗，大力推行建筑节能，在节约中求发展。实行严格的耕地保护制度，节约和集约用地，完善土地市场建设，规范建设项目用地审批，切实维护被征地农民的合法权益。严格矿山开采准入条件，规范矿业管理行为，促进矿山开发专业化、集约化。在全社会树立节约资源的观念，大力倡导绿色文明的生活方式和消费模式，引导人们养成节水、节电、节煤、节油、节粮等良好习惯。积极开展节约型机关创建活动，发挥政府在建设节约型社会中的示范作用。

　　二是大力发展循环经济。提高资源的综合开发和利用水平，重点发展循环利用型工业和建筑业、生态型循环农业。在冶金、化工、建材、轻工、电力等行业推行清洁生产，形成一批具有较高资源利用率、较低污染排放率的循环企业，建设符合循环经济发展模式的生态园区。坚决淘汰关闭浪费资源、污染环境、工艺落后的设备和企业，完善再生资源回收利用体系，力争工业固体废弃物利用率提高到80%。

　　三是加强环境治理和生态保护。坚持预防为主、综合治理的方针，坚决制止先污染后治理、边治理边污染的现象发生。从源头上控制环境污染和生态破坏，努力达到主要污染物排放总量比“十五”末减少5%。从根本上解决洋澜湖的污染问题，加强梁子湖和花马湖等湖泊的水资源保护和水体污染治理。积极推进国土绿化，打造生态屏障，建设秀美鄂州。提高新建项目环保准入门槛，落实投资项目环境影响评价和“三同时”制度，实行生态环境“一票否决”，从制度上保证经济社会的可持续发展。

（八）促进和谐社会建设。社会发展是经济发展的目标和保证。“十一五”期间，围绕建设和谐鄂州的目标，必须大力推进各项社会事业的发展，不断加强精神文明建设和民主法制建设。

　　一是切实解决好就业再就业问题。民生问题首先是就业问题，做好就业工作事关改革发展稳定的大局。坚持就业结构优化与产业结构调整有机结合，大力发展劳动密集型企业和各类所有制中小企业，发展第三产业和社区服务业，积极培育新的就业增长点。继续实施和完善财税、信贷等优惠政策，鼓励企业增加就业岗位，加强就业培训，鼓励劳动者自主创业和自谋职业。提高就业培训的针对性、实用性和有效性，使下岗职工和农村富余劳动力真正具备一技之长，不断增强就业竞争能力。

　　二是切实维护社会稳定。完善城市低保、养老、医疗、工伤、生育等社会保障制度，积极探索和推行农村最低生活保障制度，进一步完善贫困群体大病医疗救助制度和特困学生救助制度。健全最低工资保障制度，更好地改善劳资关系，逐步提高低收入劳动者工资水平，缩小不同社会群体在收入上的过大差距。妥善处理人民内部矛盾，加强信访工作，畅通诉求渠道，构建多元化矛盾纠纷调处机制。加强社会治安综合治理和平安区建设，依法严厉打击各种犯罪活动，确保人民群众安居乐业。落实企业安全生产责任制，切实加强工矿企业安全监管。加强交通、消防和特种设备安全监管工作。大力开展食品、药品、烟草、医疗卫生市场整顿。严格安全执法，遏制重特大安全事故发生，保障人民生命财产安全。

　　三是切实加强民主政治建设和精神文明建设。自觉地接受人民代表大会及其常委会的法律监督和工作监督，自觉地接受人民政协的民主监督，自觉地接受人民群众的社会监督和新闻媒体的舆论监督，认真地听取各民主党派、工商联、无党派人士的意见和建议，不断提高政府工作的水平。不断完善政务、村务、厂务公开，发展基层民主。加大依法治市力度，继续开展普法教育，努力提高全民法律意识。大力弘扬以爱国主义为核心的民族精神和以改革创新为核心的时代精神，在全社会倡导“爱国守法、明礼诚信、团结友善、勤俭自强、敬业奉献”的基本道德规范。深入开展各种形式的精神文明创建活动，使全市上下始终保持昂扬向上的精神状态。

　　四是切实加快各项社会事业发展。加快教育结构调整，重点加强义务教育特别是农村义务教育，突破性发展中等职业教育和成人教育。加快高等教育发展步伐。完善公共卫生和医疗服务体系，缓解群众“看病难、看病贵”的压力。构建覆盖全市比较完善的公共文化服务体系，满足人民群众日益增长的文化需求。加强体育设施建设，开展全民健身活动。抓好人口与计划生育工作，稳定低生育水平，加强出生人口性别比综合治理。积极推进广播电视节目上网工程、有线电视数字化工程，不断提高广播电视的服务水平。发展残疾人事业。重视和保障妇女儿童权益。积极发展老龄事业。做好侨务、民族、宗教工作。抓好地震、气象工作，增强抗灾避灾能力。提高全民国防意识，加强人民防空建设，提高民兵和预备役建设整体水平。加强军政、军民团结，创建“双拥模范城”。

　　三、2006年的工作安排

　　2006年是“十一五”规划的奠基年，也是保持经济社会发展良好势头、乘胜前进的关键年。

　　今年政府工作总的要求是：坚持以邓小平理论和“三个代表”重要思想为指导，认真贯彻落实十六届五中全会精神和市委四届八次会议精神，以科学发展观统领全局，以实施一批重点项目为抓手，以改革开放为动力，推动经济结构调整、城市功能提升和社会事业全面进步，实现“十一五”规划良好开局。

　　主要工作目标是：地区生产总值增长12%，全社会固定资产投资增长18%，社会消费品零售总额增长12%，招商引资总额增长20%，外贸出口增长10%，万元生产总值综合能耗下降4%，地方一般预算收入增长14.1%，城镇居民人均可支配收入增长9%，农民人均纯收入增长6.5%，人口自然增长率控制在5‰以内，城镇新增就业2万人以上，城镇登记失业率控制在4.5%以内。

　　实现上述目标，重点抓好以下六个方面的工作：

　　（一）认真做好“三农”工作，促进农业增效农民增收。继续推进水产、蔬菜、畜牧、林果四大支柱产业基地连片、配套和规模经营。水产业重点建设好50万亩名优水产基地。蔬菜业重点建设好省级八个万亩无公害蔬菜基地和建设好一批保鲜、储藏、加工、运输、销售企业，使蔬菜生产向集约化、产业化方向发展。畜牧业重点引导和扶持一批优质畜禽养殖小区。林果业着力建设好退耕还林、长江防护林、生态公益林工程，并重点发展一批带动力强、辐射面广的基地和企业。以社会主义新农村建设统领“三农”工作，切实抓好示范工程建设，每个区抓一个村的试点。市、区、乡（镇、街办）对试点村，将相对集中一定的财力、物力和人力，城建、交通及社会事业投资将向试点村倾斜。加强农村生态家园建设。加大扶贫开发力度。认真做好第二次全国农业普查工作，高质量完成农业普查的各项工作任务。

　　（二）推进产业结构优化升级，提高经济增长质量和效益。大力推进技术创新，抓紧研究制定推进自主创新的地方配套政策；围绕重点领域和优势产业，组织实施一批科技项目，突破一批关键技术，开发一批拥有自主知识产权的产品；加快技术创新服务体系建设，加强知识产权保护，促进创新成果不断涌现；做大做强优势产业，重点支持一批骨干企业的技术改造工作，进一步淘汰落后生产工艺，提高产品科技含量；大力推进品牌战略，增强品牌意识，抓好品牌宣传，提高鄂州产品的知名度、信誉度、美誉度和忠诚度，扩大市场影响力，支持企业做大做强主业，做优做精品牌，赢得更多客户，抢占更多市场。

　　（三）加快重点项目和园区建设，培育经济发展后劲。继续抓好“323”工程，2006年全社会固定资产投资计划65亿元，确保鄂钢冷轧板等30个项目建成投产。进一步加大招商引资力度，精心策划和组织重大招商活动。充分发挥我市地处武汉城市圈核心圈层的优势，加快与武汉的交通、产业、市场和科教对接，尽快将我市打造成为武汉城市圈特色明显、优势突出的快速增长极。大力发展园区经济，支持葛店高新区发展生物医药等优势产业，切实抓好城东工业园、花湖开发区、金刚石刀具城、樊口工业园、红莲湖旅游度假区、梁子岛生态旅游区等园区建设。采取利益分享政策，引导项目向园区集中，实现集聚效应和规模效应。

　　（四）加快民营经济和第三产业发展，增强经济发展活力。进一步激活民间投资，充分发挥我市中小企业贷款协会的作用，鼓励引导民营业主参与协会，切实解决我市民营企业融资不畅的问题；坚决打击干扰破坏民营企业经营的不法行为，切实解决环境不优的问题，使民营企业逐步成为我市经济发展的重要支撑。大力发展商贸流通业，加快建设农村贸易市场，认真组织实施“万村千乡”工程。积极争取国家支持，启动乡镇贸易超市建设，促进工业品下乡、农产品进城。积极引导房地产业健康发展，整顿和规范房地产市场秩序，发展旧房交易市场和房屋租赁市场，加强物业管理。大力发展旅游业，推进“大旅游、大产业、大市场”建设。加强社区建设，大力发展社区服务业，在方便市民生活的同时，促进三产业健康发展。

　　（五）加大基础设施建设投入，改善生产生活条件。城市建设的重点放在扩大城市规模、提升城市功能、改善发展环境、方便人民生活、增强鄂州对外形象的项目上，把有限的资金用好用活。今年计划新建、续建道路16条，改造老城区道路7条、下水道3处。续建鄂钢跨线桥、武钢鄂州球团厂铁路立交桥，维修改造凤台桥、莲花桥、滨湖桥，建设3处游园。继续实施洋澜湖综合治理，进一步改善城市环境面貌。加大交通建设力度，实施交通建设“1091工程”，即兴建10条公路、9座桥梁和1个码头，重点新建续建樊口大桥、庙岭立交桥、东葛一级公路和葛湖路。完成区乡公路收尾工作，加快实施自然村通公路进程。抓好重点集镇建设，完善基础设施，提升综合功能。

　　（六）围绕群众最关心的利益问题，真心实意为人民群众办实事办好事。千方百计扩大就业，扩大再就业优惠证发放对象范围，延长扶持政策的期限，将优惠证发放范围扩大到集体企业下岗职工。建立稳定的公共就业服务和就业再就业培训体系，对困难人员实施就业和社会保险援助。完善社保体系，依法扩大社会保险覆盖面，提高非公有制企业的社会参保率，认真解决进城务工人员、灵活就业人员的社会保障问题。增加特困救助投入，帮助城镇低收入家庭解决居住、上学、看病等特殊困难。设立一所惠民医院，为城镇低保户和农村特困户看病提供基本医疗保障。切实解决农村征地、城镇拆迁、国企改革、企业违法排污、项目建设过程中损害群众利益，以及拖欠克扣农民工工资、教育和医疗领域乱收费等问题，维护人民群众的合法权益。维护司法公正，确保法律法规严格实施。加强社会治安综合治理，依法打击各种犯罪活动，保障人民群众安居乐业。

　　各位代表，新的一年，我们将遵循“扶困、惠民、量力”的原则，继续为人民群众办好10件实事：1、筹资580万元，解决2万农村人口饮水困难及饮水安全问题；2、筹资1200万元，继续对义务教育阶段4万名城乡贫困学生实行“两免一补”；3、筹资1300万元，对农村50个排涝抗旱泵站进行维修改造；4、筹资720万元，启动农村低保，对2万农村特困人口实施基本生活保障；5、筹资500万元，继续对30个重点贫困村实施整村推进扶贫开发；6、筹资260万元，对全市9万名0—7岁的儿童实行免费计划免疫；7、设立300万元专项基金，继续对特困群体实行大病医疗救助；8、筹资240万元，提供600个公益岗位，重点解决城镇无生活来源的“零就业家庭”的就业问题；9、筹资80万元，继续实施“阳光工程”和农民技术培训工程，转移培训农村劳动力8000人；10、筹资2200万元，完成五丈港改造和洋澜湖治污一期工程。

　　四、加强政府自身建设

　　以提高行政能力为核心，切实加强政府自身建设，坚持科学执政、民主执政、依法执政，坚持权为民所用、情为民所系、利为民所谋，努力建设一个人民满意的政府。

提高决策能力。调查研究是科学决策的基础。将大部分时间与精力放在下基层调查情况、研究问题上，做到勤于沉下身子、敏于发现问题、善于剖析问题，吃透“上情”、明了“下情”、掌握实情，建立和完善深入了解民情、充分反映民意、广泛集中民智、切实珍惜民力的决策机制；注重知识积累，大力推进学习型政府建设，勤于学习现代经济、法律、政治、科技知识，提高理论素养，培养战略思维，努力提高工作的预见性和决策的科学性；牢固树立机遇意识，面对市场经济条件下稍纵即逝的发展机遇，特别是在项目建设、投资引资等工作中，抢抓机遇、勇于负责、敢于决策，避免层层请示、犹疑不决而丧失机遇、贻误发展；正确处理决策环节中民主与集中的关系，做到坚持民主决策与发挥临机决断能力相结合，努力实现行政成本最小化、行政效益最大化的决策目标。

　　提高公共服务和社会管理能力。积极顺应政治体制改革与经济体制转轨的新形势，加快转变政府职能，在履行好“经济调节、市场监管”职能的同时，更加注重“公共服务、社会管理”职能，不断提高政府的公共服务能力。牢固树立科学的发展观，坚持“五个统筹”，切实做到全面发展、协调发展、可持续发展；突出财政公共服务职能，加快建立和健全公共财政框架，在财政支出上进一步向公共产品和公共服务上倾斜，增强财政资金的公共保障能力；在配置财政资金等政府掌握的公共资源时，切实做到阳光操作、程序规范，体现公平、公正、效率，不断提高政府的公共服务水平。

　　提高落实能力。地方发展的差距、部门工作的差距，有历史、客观的原因，但更重要的是落实能力的差距。落实能力是政府行政能力的集中体现，落实的力度决定了发展的速度。进一步增强政府工作人员特别是领导干部驾驭全局、谋划全局的能力，善于将自己的工作置于全市工作大局来思考，跳出就本职工作论本职工作、就分管工作论分管工作的圈子，立足全市发展大局，切实增强把握全局的能力、洞察事物的能力和促进和谐的能力；进一步增强政府工作人员特别是领导干部解决具体问题、化解具体矛盾的能力。当前我市正处于经济体制转轨、社会结构转型的关键时期，各种矛盾和问题错综复杂，因此，必须把解决具体问题、化解具体矛盾的能力作为政府工作的重要能力。必须把自己所承担的具体事情做好、做细、做实，对看准的事、定下来的事、干起来的事，抓住不放、一抓到底；进一步增强政府工作人员特别是领导干部依法行政的能力，加强政府法制建设，加快建立权责明确、行为规范的行政执法体制，实行行政执法责任制，保证各级行政管理机关及其工作人员严格按照法定权限和程序行使职权、履行职责，切实营造一个快速、高效、优良的服务环境。加强行政效能建设，实行目标管理，不断完善督查机制，打通不落实的关节，解决好落实难的问题。

　　提高廉政建设能力。加大源头治腐力度，以“制权、管钱、用人”为重点，继续深化行政审批制度、财政管理制度、投资体制、干部人事制度四项改革，继续完善土地出让、工程项目招投标、产权交易、政府采购四项制度，继续规范行政服务中心、政府采购中心、综合招投标中心、国库集中收付中心四个中心。切实做到从制度上反腐，在程序上防腐；全面落实廉政建设责任制，加大行政监察力度，强化经济责任审计，认真落实廉政建设的各项要求。加大各种违规案件查处力度，坚决查处政府工作人员中的腐败行为；注重提高公务员队伍的素质，严格行政执法责任制度和过错追究制度。加强公务员队伍的道德修养，牢固树立正确的世界观、人生观、价值观，做到常修为政之德、常怀律己之心、常思贪欲之害，切实打造一个为民、务实、清廉的政府。

　　各位代表，过去的五年，我们万众一心、铸就辉煌；新的五年，我们信心百倍、豪情满怀。让我们在中共鄂州市委的领导下，紧紧依靠全市百万人民，以崭新的风貌、旺盛的精神、扎实的作风，开拓进取，乘势而上，为建设一个经济更加繁荣、社会更加和谐、环境更加友好、人民更加安居乐业的鄂州而努力奋斗。
